# Report of a rare case of congenital mitral valve prolapse with chronic kidney disease––reconsidered genotype–phenotypic correlations

**DOI:** 10.1002/mgg3.1558

**Published:** 2020-11-22

**Authors:** Liping Sun, Xinzhou Zhang

**Affiliations:** ^1^ Shenzhen Key Laboratory of Renal Department of Nephrology Shenzhen People’s Hospital (The Second Clinical Medical College, Jinan University; The First Affiliated Hospital, Southern University of Science and Technology) Shenzhen China

**Keywords:** chronic renal failure, *DCHS1* mutation, genotype–phenotype correlations, mitral valve prolapse

## Abstract

**Background:**

Mitral valve prolapse (MVP) is a common cardiovascular disease defined as a late systolic click or mitral valve lobes that move up into the left atrium during ventricular systole, with or without mitral insufficiency. Dachsous catherin‐related 1 (*DCHS1*) is one of the two known pathogenic genes associated with MVP. However, there is little information about the renal dysfunction caused by MVP and *DCHS1* mutations.

**Methods:**

We analyzed the genetic etiology in a rare case of 9‐year‐old boy affected by chronic renal failure with MVP. Subsequently, we constructed stable cell lines overexpressing wild‐type *DCHS1* or mutant *DCHS1* (c.8309G>A, p.R2770Q) to evaluate the influence of the *DCHS1* mutation on the proliferation, apoptosis, and autophagy.

**Results:**

Complete exome sequencing and pedigree verification revealed a mutation p.R2770Q (c.8309G>A) in exon 21 of the *DCHS1* gene carried by the patient, which may affect the DNA binding. No such mutation was detected in his parents, indicating that this was a new mutation. Potential functional impact of sequence variants was predicted using in silico prediction programs including SIFT, Polyphen2, and Condel. This variant was determined to be a pathogenic mutation that has not been reported elsewhere. Subsequently, we used a stable *DCHS1* gene‐mutated HK‐2 cell line to analyse proliferation, apoptosis, and autophagy, showed that kidney volume decreased with increasing cell death associated with a reduced proliferation.

**Conclusions:**

Our analysis revealed a heterozygous variation of *DCHS1* in a child with MVP. Our observations highlight previously unrecognized phenotypes of the currently recognized MVP genotype, including distinct chronic renal failure.

## Graphical abstract

A previously unrecognized phenotype of CKD identified in a case of MVP. *DCHS1* c.8309G>A (p.R2770Q) mutation inhibits the proliferation of renal tubular epithelial cells. The *DCHS1* mutation promotes apoptosis and autophagy in renal tubular epithelial cells. This report provides a new genotype–phenotype correlation in MVP.

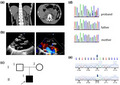

## 1 INTRODUCTION

Mitral valve prolapse (MVP) is a common cardiovascular disease defined as a late systolic click or a mitral valve lobe that shifts upward into the left atrium during ventricular systole, with or without mitral valve insufficiency (Freed et al., 1999). MVP affects 2%–3% of the general population and is the most common cause of mitral regurgitation (MR) in developed countries (Hayek et al., 2005; Nalliah et al., 2019). It is characterized by mitral valve leaf thickening and verbosity, indicating myxoid degeneration with increased lobular flexibility. Mitral myxoma is associated with abnormal stratification, characterized by loose collagen in the fibrous membrane, and damaged elastin in the atrial myocardium (Basso et al., 2015). Lobules are at least 5 mm thick in classic MVP and less than 5 mm thick in the nonclassical subtypes. Severe classic MVP is associated with arrhythmias, endocarditis, and heart failure and requires valvular surgery. Myxoid degeneration is the most common type of MVP pathology and the most common type of valve pathology that requires surgery for MR (Han et al., 2018). Studies have indicated that primary myxoid MVP is an inherited disease with an autosomal dominant and X‐linked inheritance pattern (Fulton et al., 2018), although its genetic abnormalities remain to be fully elucidated. In 2015, Durst et al. reported for the first time that the involvement of the dachsous catherin‐related 1 (*DCHS1*) gene on chromosome 11 in the pathology of myxoid MVP, among which two missense mutations (p.R2513H and p.R2330C) have been identified (Durst et al., 2015). However, the molecular genetic basis of chronic renal failure observed in some MVP patients remains unexplained. In this study, we report a case of MVP with chronic renal failure with a heterozygous *DCHS1* mutation. Furthermore, we used stable cell line with *DCHS1* mutate gene to evaluate the influence of *DCHS1* c.8309G>A (p.R2770Q) mutation on the proliferation, apoptosis, and autophagy of HK‐2 cells. Our findings indicated that the decreased kidney function may be related to increased renal cell death and decreased proliferation.

## 2 PATIENT AND METHODS

### 2.1 Ethical compliance

All subjects provided written informed consent and the study was approved by the Ethics Committee of Shenzhen People's Hospital (LL‐KY‐2019293, China).

### 2.2 Patient

A 9‐year‐old boy was admitted to the Shenzhen People's hospital with kidney failure. The laboratory results are shown in Table 1. Samples and medical history were collected with the full informed consent of the patient and his parents in accordance with the declaration on the human genome prepared by the United Nations Educational Scientific and Cultural Organization. This study was approved by.

**Table 1:** Laboratory data at presentation.

Variable	Result	Reference range
**Serum chemistry**		
Urea nitrogen (mmol/L)	26.55	2.5–7.5
Creatinine (μmol/L)	719	44–133
Cystatin C (mg/L)	3.56	0.4–1.55
Uric acid (μmol/L)	765	90–420
Glucose (mmol/L)	5.78	3.9–6.1
Total protein (g/L)	67.0	60–83
Albumin (g/L)	34.5	35–55
Cholesterol (mmol/L)	4.24	3.4–6.5
Triglyceride (mmol/L)	1.46	0.4–2.3
Serum potassium (mmol/L)	4.41	3.5–5.5
Serum sodium (mmol/L)	143.4	135–145
Serum chlorine (mmol/L)	106.5	96–110
Serum calcium (mmol/L)	2.17	2.05–2.55
Serum phosphorus (mmol/L)	2.10	0.8–1.5
Carbon Dioxide Combining Power (mmol/L)	15.6	22–34
Parathyroid hormone (pg/mL)		
Urine test		
Specific gravity	1.013	1.01–1.03
Urinary protein	2.0	0–0.3
Urine glucose	—	<5
Urine ketone bodies	—	<0.6
Urine bilirubin	—	—
Urinary occult blood	50	<10

**FIGURE 1:**
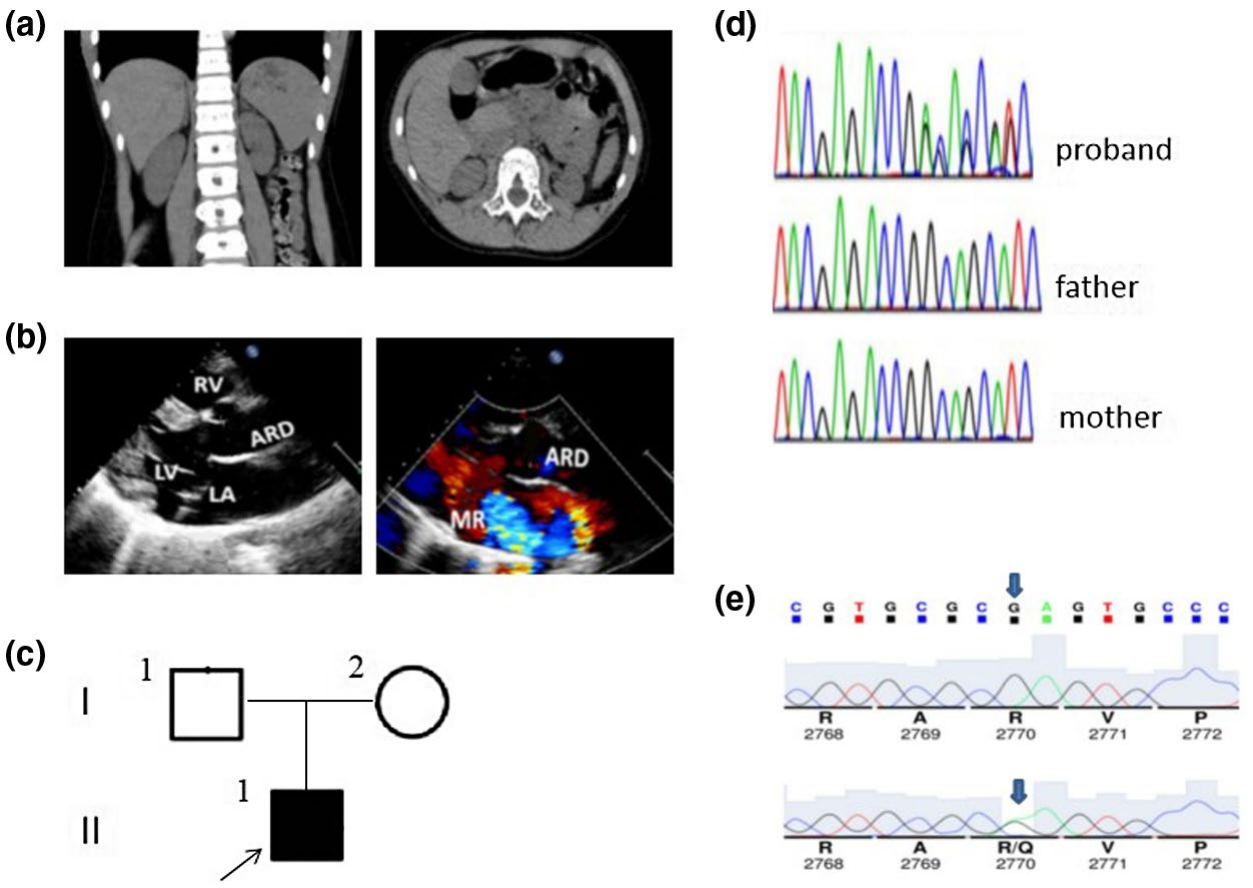
Clinical, radiological, and genetic findings of the patient. (a) CT examination indicates bilateral renal atrophy, especially the left kidney. (b) Ultrasonic cardiogram (c) Pedigree chart of the patient's family. I1, I2, and II1 represent the father, mother, and patient, respectively, and the arrows represent the proband. Black represents DCHS1 mutation. (d) Genetic analysis shows a heterozygous *DCHS1* mutation, c.8309G>A was identified in the patient, but not in her parents. (e) Sequence chromatograms of the rare (p.R2770Q) missense in silico‐predicted deleterious variants identified in this study.

Urine analysis revealed the sample was occult blood + and protein ++. Serological analysis revealed elevated urea nitrogen, creatinine, and cystatin C (26.55 mmol/L, 719.0 μmol/L, and 3.56 mg/L, respectively). In addition, the CT examination of the urinary system revealed that the patient's left and right kidneys were 53 × 22 mm and 61 × 27 mm, respectively, indicating bilateral renal atrophy (Figure 1a). In addition, except for continuous nocturia, there were no obvious abnormalities on physical examination. In terms of treatment, the patient began continuous peritoneal dialysis after admission to relieve renal deterioration.

The patient had occasional chest tightness, palpitations (heart rate 113 bpm), no dyspnea, chest pain or discomfort, and exercise was not restricted under normal conditions. Hypertension, hyperlipidemia, pulmonary hypertension, and no atrial fibrillation. The results of echocardiography indicated a slight increase in left ventricular diameter (left ventricular end‐diastolic diameter 56 mm and end‐systolic diameter 34 mm), with a left ventricular ejection fraction of 60%. Mitral thickening and prolapse with severe regurgitation and tricuspid thickening and prolapse with mild regurgitation were also observed. The shape of the aortic sinus was acceptable, with an internal diameter of 34 mm. The calculated Z‐value of the internal diameter of the aortic sinus was 3.1. The echocardiographic manifestations of the patient are shown in Figure 1b.

The parents of the patient were not consanguineous and were in good health. The parents had a negative family history of heart and kidney disease. The mother had no special pregnancy history, and the patient was delivered naturally at term, with a birth weight of 2.9 kg, and no history of neonatal asphyxia or infection. No obvious abnormalities were detected in the outer court genetic metabolic screening and microarray comparative genomic hybridization analysis (Affymetrix Cytogenetics whole genome––2.7 M array).

### 2.3 Methods

#### 2.3.1 Materials

Human tubular epithelial cells HK‐2 were purchased from the America Tissue Culture Collection. The reverse transcription and SYBR Green qRT‐PCR kits were purchased from TOYOBO, Japan.

The following antibodies were used in this study: rabbit anti‐caspase3 antibody (1:1,000, 8G10, 9665; Cell Signaling Technology), rabbit anti‐cleaved‐PARP antibody (Asp214) (1:1,000, D6X6X, 94885, Cell Signaling Technology), rabbit antibody to beclin‐1 antibody (1:1,000, D40C5, 3495, Cell Signaling Technology), rabbit anti‐LC3B antibody (1:2,000, EPR18709, ab192890, Abcam), rabbit anti‐DCHS1 antibody (1:1,000, Ab203690; Abcam), rabbit anti‐cyclin D1 antibody (1:200, SP4, ab16663, Abcam), rabbit anti‐CDK4 antibody (1:2,000, EPR17525, ab199728, Abcam), mouse anti‐p27 antibody (1 μg/ml, SX53G8, ab193379, Abcam), and rabbit anti‐LC3B antibody (1 μg/ml, EPR18709, ab192890, Abcam).

#### 2.3.2 Detection of genetic mutation

Genomic DNA was extracted from the peripheral blood of the patient and his parents in accordance with standard procedures. The samples were then sequenced using Total Exon capture chips with the SureSelect Human All Exon Target Enrichment kit (Agilent Technologies), followed by next‐generation sequencing using the HiSeq 2500 Illumina platform (Illumina). The ABI Prism 3130XL DNA analyzer (Life Technologies GmbH, Darmstadt, Germany) was used to read the sequence. The mutations detected were further validated by bidirectional Sanger sequencing of the DNA samples obtained from the patient and his parents.

Because in silico prediction tools may give different result, a good rule is to consider a prediction only when Polyphen2 (http//genetics.bwh.harvard.edu/pph2/) SIFT (http//sift.jcvi.org/), and the CONsensus DELeteriousness score of missense mutations (Condel) disease prediction tools give the same result. The pathogenicity of the sequence variants was predicted using in silico prediction programs including SIFT, Polyphen2, and Condel.

#### 2.3.3 Cell culture and generation of stable DCHS1 mutation cells

HK‐2 cells were cultured in Dulbecco's modified Eagle's medium (DMEM) supplemented with 8% fetal bovine serum at 37°C under 5% CO_2_. After 3 days, when the cells had reached confluent growth, the cells were digested with 0.25% trypsin and passaged.

*DCHS1* (GenBank, NG_033858.2, NM_003737.4) was cloned to construct HK‐2 (*DCHS1*‐WT) stable cell line, *DCHS1* (c.8309G>A, p.R2770Q) was cloned to construct HK‐2 (*DCHS1*‐Mut) stable cell line, and these two cell line were constructed by Hanbio Technology Co., Ltd. The expression of *DCHS1* was detected by qPCR and Western blot analysis.

#### 2.3.4 qPCR

Total RNA was extracted from cells and serum using TRIzol reagent and an RNeasy Plus Mini Kit (Qiagen, Valencia, CA, USA) according to the manufacturer's instructions. After reverse transcription using a High‐Capacity cDNA reverse Transcription Kit (Applied Biosystems) as per the manufacturer's protocol, cDNA was used as the template for PCR amplification of *DCHS1* and GAPDH as the internal reference using the following primers synthesized by Shanghai Shenggong: DCHS1_F 5'‐AGCTGTGAATCCTTGAGGCCA‐3'; Probe FAM‐AGGTGCTGATGT GGAGTGTTGGGG‐MBG; and DCHS1_R 5'‐GGTCAGCTGCAGCCACTG‐3'; GAPDH_F 5'‐TGACTTCAACAGCGACACCCA‐3'; GAPDH_R 5'‐CACCCTGTT GCTGTAGCCAAA‐3'. QuantiFast SYBR Green real‐time PCR Mastermix (Qiagen) was used for qRT‐PCR assays on the CFX Connect 96‐well real‐time PCR system (bio‐rad Laboratories) under the following thermocycling conditions: 95°C for 15 s, 60°C for 30 s, and 72°C for 30 s; 40 cycles. *Dchs1* mRNA expression was quantified using the 2^−ΔΔCt^ method and normalized to the gene expression of GAPDH.

#### 2.3.5 Western blot analysis

Total proteins were extracted and the protein concentration was determined using the BCA method. After denaturation in boiling water for 10 min, 40 g of total protein was separated by SDS‐PAGE (10% gel) and then, transferred to polyvinylidene fluoride membranes. The membrane was then blocked in 5% nonfat dried milk solution at room temperature for 30 min before incubation with primary detection antibodies overnight at 4°C. After washing with TBST, the membrane was then incubated secondary detection antibodies at 37°C for 2 hr. After washing with TBST, protein bands were visualized by incubation with ECL solution for 5 min. The membranes were then developed with WesternBright ECL (Advansta, Menlo Park, CA, USA) for imaging. Image lab software was used to acquire and analyze imaging signals.

#### 2.3.6 Cell proliferation assay

Cell proliferation was assayed using the Cell Count Kit‐8 method (CCK‐8; DojinDo Kumamoto, Japan) according to the manufacturer's instructions. Briefly, cells were seeded in 96‐well plates (5 × 10^3^/well) and cultured as described in Section 2.2.3. After plating for 24, 48, and 72 hr, CCK‐8 solution (10 μl) was added to each well and the plates were incubated at 33°C for a further 2 hr. The optical density (OD) at 450 nm was measured for each well using a miniature plate reader (Multiskan GO; Thermo Scientific).

#### 2.3.7 Flow cytometric analysis of cell apoptosis

Apoptosis was measured by flow cytometric analysis of annexin V‐FITC/PI double‐staining. Cells cultured under experimental conditions were harvested and resuspended in 100 μl annexin V binding buffer. After the addition of 5 μl annexin V‐FITC, cells were incubated in the dark at 4°C for 15 min. The rate of apoptosis of the cells was then analyzed using a FACSort flow cytometer.

#### 2.3.8 Analysis of autophagy dynamics in transfected cells

The dynamic process of autophagy was analyzed in cultured HK‐2 cells expressing mRFP‐GFP‐LC3 as d l. The rationale of this method is that acid‐sensitive GFP is quenched in the low pH lysosomal environment, whereas acid‐insensitive RFP is more stable and maintained. Thus, co‐localization of RFP fluorescence with GFP in a particle indicates an autophagosome. HK‐2 cells were transiently transfected with mRFP‐GFP‐LC3 (ptfLC3, Addgene plasmid 21074). After treatment, the cells were fixed with 4% paraformaldehyde for fluorescence microscopy (Zeiss 780 upright confocal microscope). The numbers of GFP‐LC3 puncta per cell and RFP‐LC3 puncta per cell were counted separately using ImageJ. The number of autophagosomes was indicated by GFP dots and the number of autolysosomes was obtained by subtracting the number of GFP dots from the number of RFP dots. The number of autolysosomes was further divided by the total number of RFP dots to determine the autophagic flux rate.

For immunofluorescence, HK‐2 cells were inoculated into 24‐well plates at a density of 10^5^ cells per well. Cells were fixed with 4% polyformaldehyde and permeabilized in 0.25% Triton X‐100 for 10 min. After blocking, the cells were cultured overnight at room temperature and incubated with primary antibodies overnight at 4°C. The cells were then incubated with secondary antibodies for 1 hr at room temperature. Images were obtained either with a confocal laser scanning microscope (Zeiss LSM 510) or a Nikon TE2000U inverted microscope equipped for widefield fluorescence detection and MetaMorph (Universal Imaging) acquisition software. Where noted, images were post‐processed with AutoDeblur/AutoVisualize (MediaCybernetics) deconvolution software.

#### 2.3.9 Statistical analysis

All tests were performed at least three times. SPSS 13.0 software was used for statistical analysis. Quantitative data were expressed as mean ± standard deviation. One‐way ANOVA was used for statistical evaluation, and differences between groups were evaluated using Fisher's LSD test or Dunnett's T3 tests. *p* < 0.05 was considered statistically significant.

## 3 RESULTS

### 3.1 Gene mutation characteristics

Bioinformatics analysis of the patient revealed the presence of *DCHS1* gene 21 exon containing c.8309G>A (p.R2770Q) mutation that was not detected in the parents (Figure 1c,d). Clinical significant variant(s) were not identified in the patient, and deep intronic variants were not evaluated which is a limitation of whole‐exome sequencing. In addition, the child had typical facial features, with no intellectual disability, hearing loss, bone and limb abnormalities, or endocrine abnormalities, and the diagnosis of Van Maldergem syndrome type 1 (OMIM: 601390) was excluded. The rare missense variant (p.R2770Q) was identified in one heterozygote patient characterized by a G to A substitution (c.8309G>A) resulting of a glutamine instead of an arginine at position 2,770 of the protein (Figure 1e). Then, we identified the additional in silico‐predicted deleterious variants p.R2770Q.

### 3.2 Establishment of stable *DCHS1*‐mutation cell line

Phenotypic analysis of *DCHS1* mutant mice revealed that *DCHS1* is required for growth, branching, and cell survival in early kidney development. One of the most obvious phenotypes of *DCHS1* mutation is a reduction in kidney size. To investigate the cellular basis of kidney size reduction caused by *DCHS1* mutation, we analyzed the effect of *DCHS1* mutation on proliferation and apoptosis of renal tubular epithelial cells.

**FIGURE 2:**
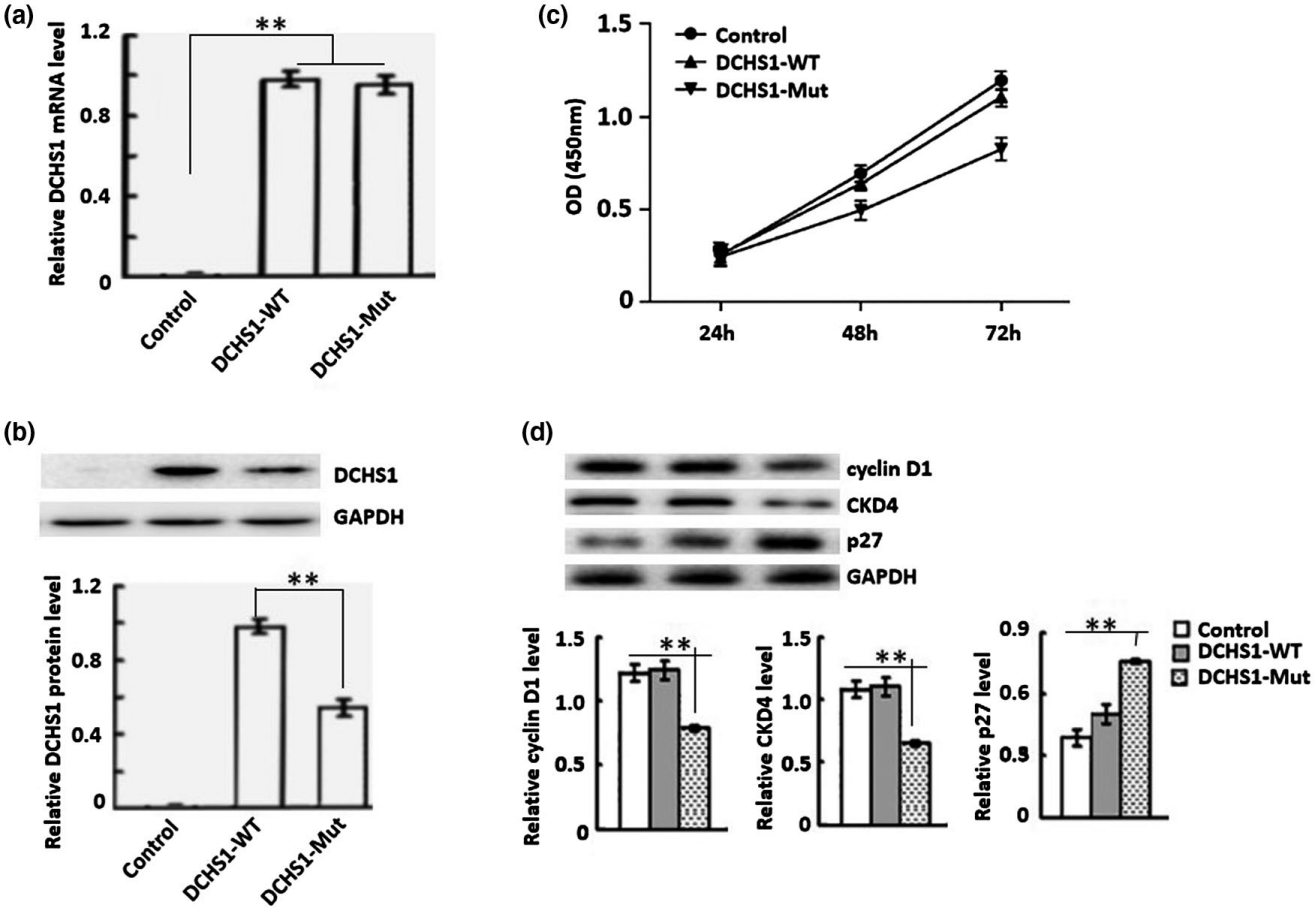
Efficiency of *DCHS1* c.8309G>A (p.R2770Q) mutation in cultured HK‐2 cells. (a) Efficiency of *DCHS1* c.8309G>A (p.R2770Q) mutation in HK‐2 cells was determined by qRT‐PCR. Control: wild‐type; *DCHS1*‐WT: *DCHS1* was cloned to construct HK‐2 stable cell line; *DCHS1*‐Mut: *DCHS1* (c.8309G>A, p.R2770Q) was cloned to construct HK‐2. (b) Western blot analysis of the efficiency of *DCHS1*‐Mut: in HK‐2 cells. (c) OD values of the three groups of cells at 24, 48, and 72 hr were measured by CCK‐8 assay. Western blot analysis of the expression of cyclin D1 and CDK4 protein. **p < 0.01.

We constructed and screened human tubular epithelial cell lines with stable expression of *DCHS1* and *DCHS1* c.8309G>A (p.R2770Q) mutation. The expression levels of *DCHS1* were measured by qPCR (Figure 2a) and Western blot (Figure 2b) analyses. The results showed that the expression of *DCHS1* mRNA and DCHS1 protein was increased in the *DCHS1*‐WT and *DCHS1*‐Mut stable cell line group compared with the control. The expression of *DCHS1* c.8309G>A (p.R2770Q) mutant protein was ~40% less than wild type, but no significant change in mRNA levels, suggesting that the *DCHS1* variants reduce protein stability.

### 3.3 Mutation of *DCHS1* inhibits HK‐2 cells proliferation

At 24, 48, and 72 hr after stable transfection with *DCHS1* shRNA, OD values were significantly lower than those of the normal or negative controls (Figure 2c). In addition, the protein levels of CCND1 (cyclin D1) and CDK4 were decreased, while CDKN1B (p27) expression was increased during podocytosis of stably transfected renal tubular epithelial cells (Figure 2d).

The cell proliferation of *DCHS1*‐Mut cells was analyzed by a CCK‐8 assay. Compared with Control cells, the proliferation of *DCHS1*‐WT cells was increased, whereas the *DCHS1*‐Mut cells were decreased significantly (Figure 2c). In addition, the protein levels of CCND1 (cyclin D1) and CDK4 were decreased, while CDKN1B (p27) expression was increased in *DCHS1*‐Mut cells (Figure 2d).

### 3.4 *DCHS1* mutation promotes apoptosis of renal tubular epithelial cells

**FIGURE 3:**
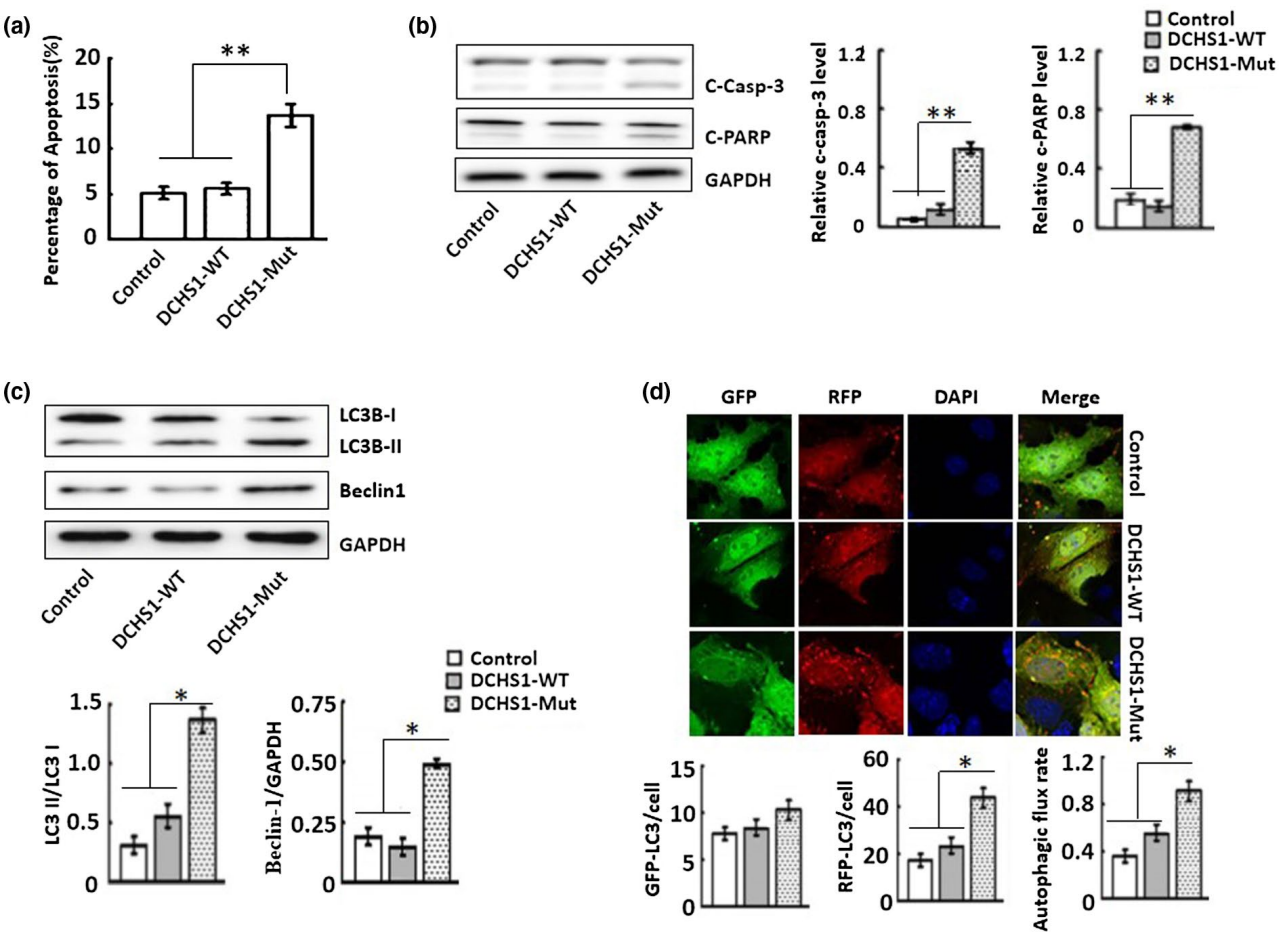
*DCHS1*‐Mut promotes HK‐2 cell apoptosis and autophagy. (a) Flow cytometric analysis of apoptotic cells was quantified by Annexin V‐FITC/PI double‐staining. (b) Western blot analysis of the presence of cleaved‐Caspase‐3 and cleaved‐PARP. (c) Western blot analysis of the expression of Beclin1, LC3B‐I, and LC3B‐II. (d) Immunofluorescence analysis of LC3B expression. LC3B was labeled green with Alexa Fluor 488‐conjugated secondary antibodies, cell nuclei were stained blue with DAPI. Scale bars: 50 μm. *p < 0.05, **p < 0.001.

The apoptosis rate of *DCHS1*‐Mut cells was increased (Figure 3a). In accordance with these results, western blot analysis showed that the levels of cleaved‐Caspase‐3 and cleaved‐PARP in *DCHS1*‐Mut cells were significantly higher than those in the normal or *DCHS1*‐WT cells (Figure 3b).

### 3.5 *DCHS1*‐Mut induces autophagy in renal tubular epithelial cells

In *DCHS1*‐Mut cells, the expression of Beclin1 protein and the conversion of the cytoplasmic form of LC3B‐I to its lipid membrane binding form LC3B‐II were also increased (Figure 3c). Similar results were observed by immunofluorescence analysis (Figure 3d), which showed granular, punctate staining in HK‐2 cells. Compared with the control and *DCHS1*‐WT group, increased numbers of LC3B puncta were detected in HK‐2 tubules transfected with *DCHS1* c.8309G>A (p.R2770Q) mutation.

## 4 DISCUSSION

MVP refers to mitral valve leaflets (single leaf or anterior and posterior leaflets) that are abnormally lower than the level of the mitral annulus during ventricular systole and detached into the left atrium. It is a common heart valve malformation, with some genetic predispositions, and mainly pathological changes. With the exception of a few patients who may be complicated by severe MR, congestive heart failure, infective endocarditis, and/or moderate stroke, most patients with MVPs are considered to have a good prognosis without the need for medication or surgical intervention (Pocock et al., 1984). However, cases of myocardial ventricular arrhythmia (MVA) and sudden cardiac death (SCD) in patients with MVP are often reported. The risk of MVP developing into MVA increases by 50%–60%, and the MVP sudden death rate is as high as 0.4%–2% (Fulton et al., 2018). In recent years, the risk factors of MVA and SCD in MVP patients have become a hot topic in the field of MVP research. Studies have revealed autosomal dominant inheritance and X‐linked inheritance patterns in primary mucinous MVP. *DCHS1* and *FLNA* are two pathogenic genes that have been discovered so far.

The causal relationship between *DCHS1* mutation and chronic renal failure has not yet been elucidated. Mao et al. analyzed the phenotype of *DCHS1* mutant mice and made comparisons with *Fat4* mutant mice to determine the functions of these genes in multiple organs, including ear, kidney, bone, intestine heart, and lungs (Mao et al., 2016). It was found that *DCHS1* and *Fat4* single and double mutants had similar phenotypes throughout the body. Both DCHS1 and Fat4 were found to function as ligand‐receptor pairs during mouse development, and the role of DCHS1‐Fat4 signaling on multiple organs was determined. DCHS1 and Fat4 are mainly expressed in mesenchymal cells of multiple organs. Mutation of either of these genes increased the expression of the gene encoded by the other gene. Some reports of these phenotypes indicate that the DCHS1–Fat4 signaling pathway affects planar cell polarity (Durst et al., 2015; Zakaria et al., 2014). In addition to the occurrence of cysts in the kidneys of newborns, the requirements for *DCHS1* and Fat4 for growth, branching, and cell survival in early kidney development were identified and described.

However, the molecular genetic basis of chronic renal failure observed in some MVP patients remains unexplained. To investigate the role of *DCHS1* mutation in renal tubular epithelial cells, we established stable *DCHS1* c.8309G>A (p.R2770Q) mouse tubular epithelial cell lines (*DCHS1*‐Mut) and revealed that *DCHS1*‐Mut cells disrupted the proliferation, apoptosis, and autophagy of renal tubular epithelial cells.

In this study, we observed a decrease in kidney size and branching during early kidney development in a patient with a mutation in the *DCHS1* gene. To investigate the mechanism of renal contraction, we examined the effect of *DCHS1*‐Mut on podocyte proliferation using CCK8. Our results showed increased in OD values of *DCHS1*‐WT cells, whereas proliferation were significantly decreased in *DCHS1*‐Mut cells. In addition, our research also showed that *DCHS1*‐Mut reduced the expression of cyclin D1 and CDK4 and increased the expression of p27. These results indicated that the *DCHS1* c.8309G>A (p.R2770Q) mutation affected cell proliferation by regulating the expression of cell cycle regulators (cyclin D1, CDK4, and p27), which could be attributed to an alteration in *DCHS1* function caused by the mutation.

Tubular epithelial cell apoptosis is considered one of the key steps in the development of progressive nephropathy (Schiffer et al., 2001). In this study, we conducted flow cytometric analysis of annexin V‐FITC/PI staining to determine the effect of *DCHS1* c.8309G>A (p.R2770Q) mutation on apoptosis. We found that the apoptosis rate of *DCHS1*‐Mut was higher than that of the two groups of controls. Similarly, the levels of cleaved‐Caspase‐3 and cleaved‐PARP in *DCHS1*‐Mut cells were higher than those in the two control groups. These results indicate that renal tubular epithelial cell apoptosis is promoted by the mutation of the *DCHS1* gene. In addition, our study showed that *DCHS1* c.8309G>A (p.R2770Q) mutation also induces autophagy. In *DCHS1*‐Mut cells, the expression of becn1 (beclin1) and LC3B (LC3B‐II) both increased. Autophagy is a lysosomal degradation pathway by which damaged proteins and organelles are degraded to maintain intracellular homeostasis (Mizushima & Komatsu, 2011); however, excessive autophagy causes cell death (El‐Khattouti et al., 2013). Therefore, we speculate that the *DCHS1* mutations, promote podocyte apoptosis and enhance renal tubular cell autophagy, leading to chronic renal failure.

In conclusion, a previously unknown *DCHS1* c.8309G>A (p.R2770Q) mutation was identified in the case investigated in this study. Our observations reveal a previously undiscovered phenotype of the currently recognized MVP genotype, including obvious chronic renal failure. This report challenges the current belief in genotype–phenotype relationships. Furthermore, the *DCHS1* mutation inhibited cell proliferation and induce apoptosis and autophagy.

## CONFLICT OF INTEREST

The authors declare no conflict of interest.

## AUTHOR CONTRIBUTIONS

L.P.S. conceived and designed the study. L.P.S. wrote the first draft of the manuscript. L.P.S. and X.Z.Z. edited the manuscript. All authors have read and approved the published version of the manuscript.

## ETHICAL STATEMENT

All subjects gave their informed consent for inclusion before they participated in the study. The study was conducted in accordance with the Declaration of Helsinki, and the protocol was approved by the Ethics Committee of Shenzhen People's Hospital (LL‐KY‐2019293).

## Data Availability

The data supporting the conclusions of this article will be made available by the authors, without undue reservation, to any qualified researcher.
